# Early Post-transcatheter Aortic Valve Implantation (TAVI) Haemodynamics With SAPIEN Balloon-Expandable and Evolut Self-Expanding Valve Platforms: A Retrospective Single-Centre Cohort Study

**DOI:** 10.7759/cureus.116667

**Published:** 2026-09-21

**Authors:** Behzad Masood, Mamoon Ahmed, Syed M Usman, Afzaal Aleem Khan, Saba Ayub

**Affiliations:** 1 Cardiology, Sheffield Teaching Hospitals NHS Foundation Trust, Sheffield, GBR; 2 Internal Medicine, Sheffield Teaching Hospitals NHS Foundation Trust, Sheffield, GBR; 3 Acute Medicine, Russells Hall Hospital, Dudley, GBR; 4 Internal Medicine, University Hospitals of North Midlands NHS Trust, Stoke-On-Trent, GBR

**Keywords:** aortic stenosis (as), balloon-expandable valves, self-expanding valves, self-expanding vs. balloon-expanding valve, transcatheter aortic valve implantation (tavi), transcatheter aortic valve replacement (tavr)

## Abstract

Introduction

Balloon-expandable valves (BEVs) and self-expanding valves (SEVs) have different frame architectures, leaflet positions and distinct deployment mechanics that may influence prosthetic haemodynamics. We compared early echocardiographic gradients after transcatheter aortic valve implantation (TAVI) between SAPIEN BEV and Evolut SEV platforms in a routine-practice cohort spanning several device generations.

Materials and methods

We retrospectively analysed 665 TAVI procedures: 250 BEV procedures using SAPIEN 3 variants and 415 SEV procedures using Evolut variants. The primary endpoint was the early post-TAVI echocardiographic mean transvalvular gradient obtained within three months of TAVI. Secondary endpoints were absolute and relative gradient reductions, achievement of a post-TAVI mean gradient <10 mmHg, and a residual mean gradient ≥20 mmHg. Because the continuous variables were skewed, they were compared using two-sided Mann-Whitney U tests. Results were reported as medians with interquartile ranges, with bootstrap 95% confidence intervals (CIs) for differences in medians. Categorical variables were compared using Pearson chi-square tests without continuity correction when expected counts were adequate and Fisher's exact tests when cell counts were small. Paired pre- and post-TAVI measurements were compared using Wilcoxon signed-rank tests. Multivariable ordinary least-squares regression with HC3 heteroscedasticity-robust standard errors was used to adjust the post-TAVI gradient for the baseline gradient and available prespecified covariates.

Results

Complete pre- and post-TAVI gradients were available for 664 procedures (249 BEV and 415 SEV). The median post-TAVI gradient was 8.4 (5.85-11.63) mmHg with SEV and 10.0 (7.93-13.90) mmHg with BEV (median difference, -1.60 mmHg; bootstrap 95% CI: -2.97 to -1.00; Mann-Whitney U test: p < 0.001). The median absolute gradient reduction was also greater with SEVs (36.0 vs 32.0 mmHg; median difference, 4.0 mmHg; p < 0.001), as was the relative gradient reduction (81.52% vs 76.19%; median difference, 5.33 percentage points; p < 0.001), although SEVs had a higher pre-procedural baseline transvalvular gradient. A post-TAVI mean gradient <10 mmHg was recorded in 61.2% of SEV procedures compared with 43.4% of BEV procedures (risk ratio 1.41; p < 0.001). Residual gradients of ≥20 mmHg were uncommon and did not differ between the two groups (6.0% in SEV procedures vs 5.6% in BEV procedures; p = 0.831). After adjustment, in 575 completed cases, SEV use remained associated with a 1.48 mmHg lower post-TAVI mean gradient (95% CI: -2.42 to -0.55; p = 0.0019).

Conclusions

In this cohort, Evolut SEV procedures were associated with a modestly lower early post-TAVI mean gradient than SAPIEN BEV procedures. The magnitude of this association, and its interpretation alongside baseline gradient, anatomy, and other device-selection factors, can inform further comparative evaluation. No clinical outcomes or valve-durability measures were assessed.

## Introduction

Aortic stenosis is the most common primary valvular lesion requiring surgical or transcatheter intervention in Europe and North America, and severe symptomatic disease is associated with a poor prognosis, particularly when left-ventricular dysfunction develops [1]. Transcatheter aortic valve implantation (TAVI), also called transcatheter aortic valve replacement, has shifted from a treatment primarily used in patients at prohibitive or high surgical risk to an established option across a broad spectrum of risk groups, including those at low surgical risk [2]. As the procedure is offered to patients with longer life expectancy, the haemodynamic performance of the implanted valve, the risk of prosthesis-patient mismatch, and planning for future valve interventions have become increasingly relevant.

The VARC-3 consensus definitions reflect the relevance of residual transvalvular gradient as a measure of prosthetic valve performance by incorporating mean transvalvular gradient alongside other echocardiographic parameters to assess haemodynamic valve performance and valve deterioration [3]. In particular, a mean gradient of <20 mmHg forms part of the criteria for intended valve performance, highlighting the importance of minimising residual obstruction following TAVI [3].

Current guidelines emphasise multidisciplinary assessment and individualised valve selection according to age, life expectancy, anatomy, surgical risk, and the anticipated functional performance of the prosthesis [4,5]. Randomised trials in intermediate- and low-risk populations have further expanded the evidence base for both balloon-expandable valve (BEV) and self-expanding valve (SEV) systems [6-8].

Although both platforms relieve obstruction from severe aortic stenosis, BEVs and SEVs differ in frame architecture, leaflet position, and deployment mechanism [9]. BEVs generally use an intra-annular frame and deploy via balloon expansion. In contrast, many SEV systems position the functional leaflets supra-annularly and continue to expand after deployment [9]. A supra-annular leaflet position may provide a larger effective orifice area for a given annular size, which could benefit patients with a small annulus or a high risk of prosthesis-patient mismatch [10]. Valve selection, however, must also consider anatomy, conduction-system considerations, coronary access, and implantation depth.

Randomised and observational comparisons have generally reported differences in haemodynamic performance between the two platforms, although the size of the difference varies with valve generation and patient anatomy. The CHOICE trial compared early-generation BEV and SEV systems, and a subsequent follow-up showed a larger prosthetic valve area and lower trans-prosthetic gradients with the SEV [11,12]. Propensity-matched and anatomy-specific studies have reported similar findings, including lower gradients and less prosthesis-patient mismatch with supra-annular SEVs, particularly in patients with small annuli [13-15]. A randomised comparison of ACURATE neo and SAPIEN 3 further demonstrated that haemodynamic performance differs between valve platforms; however, in this case, the self-expanding ACURATE neo did not meet non-inferiority compared to the balloon-expandable SAPIEN 3 device in terms of early safety and clinical efficacy outcomes, highlighting the importance of platform-specific haemodynamic performance [16].

More recent evidence includes the SMART randomised trial, which compared an Evolut supra-annular SEV with a SAPIEN BEV in patients with small aortic annuli and reported lower follow-up gradients with the SEV [17].

In this study, we compared early post-TAVI haemodynamic performance between BEVs and SEVs in a large, real-world TAVI cohort to provide complementary routine-practice data from a broad, single-centre cohort spanning several generations, while retaining the limitations of a non-randomised registry analysis. Our hypothesis was that SEVs would be associated with a lower post-TAVI mean gradient and a greater proportional reduction from baseline. We therefore examined early post-TAVI echocardiographic haemodynamics between the two platform families and quantified measured-covariate-adjusted associations. We performed an exploratory comparison of contemporary Evolut FX/FX+ and SAPIEN 3 Ultra RESILIA valves. The study addressed surrogate haemodynamic endpoints and was not designed to evaluate long-term clinical outcomes or valve durability.

## Materials and methods

Study design and population

This retrospective, single-centre, non-randomised observational comparative cohort study included 665 individual TAVI procedures that were performed at Northern General Hospital, Sheffield, United Kingdom, between February 2017 and November 2025. The work was approved as registered service evaluations (Clinical Effectiveness Unit 13047, 13048); as a service evaluation using anonymised routine data, it required neither Research Ethics Committee review nor individual consent under the UK Health Research Authority framework and complied with the Declaration of Helsinki. Patients were grouped by implanted platform: BEV procedures used SAPIEN 3, SAPIEN 3 Ultra, or SAPIEN 3 Ultra RESILIA, whereas SEV procedures used Evolut R, Evolut PRO, Evolut PRO+, Evolut FX, or Evolut FX+. Reporting was conducted in accordance with STROBE recommendations [18].

Endpoints

Early post-TAVI haemodynamics were assessed on the first transthoracic echocardiography performed within three months of the procedure. The primary endpoint was the early post-TAVI echocardiographic mean transvalvular gradient. Secondary endpoints included absolute gradient reduction (pre-TAVI gradient minus post-TAVI gradient), relative gradient reduction (percentage reduction from baseline), a post-TAVI mean gradient <10 mmHg, and a residual gradient ≥20 mmHg.

Statistical analysis

Continuous variables were reported as medians with interquartile ranges because the observed distributions were skewed; categorical variables were reported as counts and percentages. Two-sided Mann-Whitney U tests were used for independent continuous comparisons because the groups were independent and normality could not be assumed. Unadjusted continuous effects were expressed as differences in medians for SEV versus BEV, with 95% confidence intervals (CIs) obtained from 4,000-replicate percentile bootstrap procedures. Pearson chi-square tests were used for categorical comparisons when expected cell counts were adequate; two-sided Fisher's exact tests were used when expected cell counts were sparse. Paired pre- and post-TAVI gradients were compared using two-sided Wilcoxon signed-rank tests. Risk ratios with 95% CIs were calculated for binary haemodynamic thresholds.

For the primary adjusted analysis, we fitted an ordinary least-squares model with post-TAVI mean gradient as the dependent variable and SEV versus BEV as the main exposure. The model adjusted for pre-TAVI gradient, sex, weight, logistic EuroSCORE, diabetes, chronic obstructive pulmonary disease, previous percutaneous coronary intervention, atrial fibrillation, and previous transient ischaemic attack or stroke. HC3 heteroscedasticity-robust standard errors were used to reduce sensitivity to unequal residual variance. The regression coefficient for the valve platform was interpreted as an adjusted mean association and not as a causal treatment effect. Complete-case analysis was used for the adjusted models, with no imputation. Because the pre-TAVI gradient was included as a covariate, the adjusted coefficient for absolute gradient reduction was algebraically the exact negative of the post-TAVI gradient coefficient and was therefore not presented as an independent model.

In the contemporary-generation sensitivity analysis comparing SAPIEN 3 Ultra RESILIA with Evolut FX/FX+, the two-sided Mann-Whitney U test and bootstrap CI were interpreted as separate inferential summaries. The Mann-Whitney U test assessed rank-based distributional separation between valve groups, whereas the bootstrap CI quantified uncertainty around the direct difference in sample medians. The contemporary-generation analysis was interpreted as exploratory and was not subject to multiplicity adjustment.

All tests were two-sided, and p < 0.05 was considered statistically significant. Analyses were performed in Python 3.12.13 using pandas 2.2.3 (Python Software Foundation, Beaverton, OR, USA), NumPy 2.3.5 (NumPy Project (NumFOCUS, Inc.), Austin, TX, USA), and SciPy 1.17.0 (SciPy Project (NumFOCUS, Inc.), Austin, TX, USA).

## Results

Study population

Of the 665 procedures, 415 (62.4%) used SEVs, and 250 (37.6%) used BEVs. The valve-specific counts were Evolut R (n = 48), Evolut PRO (n = 89), Evolut PRO+ (n = 122), Evolut FX (n = 95), Evolut FX+ (n = 61), SAPIEN 3 (n = 25), SAPIEN 3 Ultra (n = 143), and SAPIEN 3 Ultra RESILIA (n = 82). After exclusion of one procedure with a missing post-TAVI gradient, 664 procedures, comprising 415 SEVs and 249 BEVs, formed the primary haemodynamic analysis cohort. The adjusted analysis included 575 complete cases; 89 procedures with complete paired gradients were excluded from the regression analysis because of missing or invalid data for at least one prespecified covariate. Figure 1 shows the study population and analysis cohorts.

**Figure 1:**
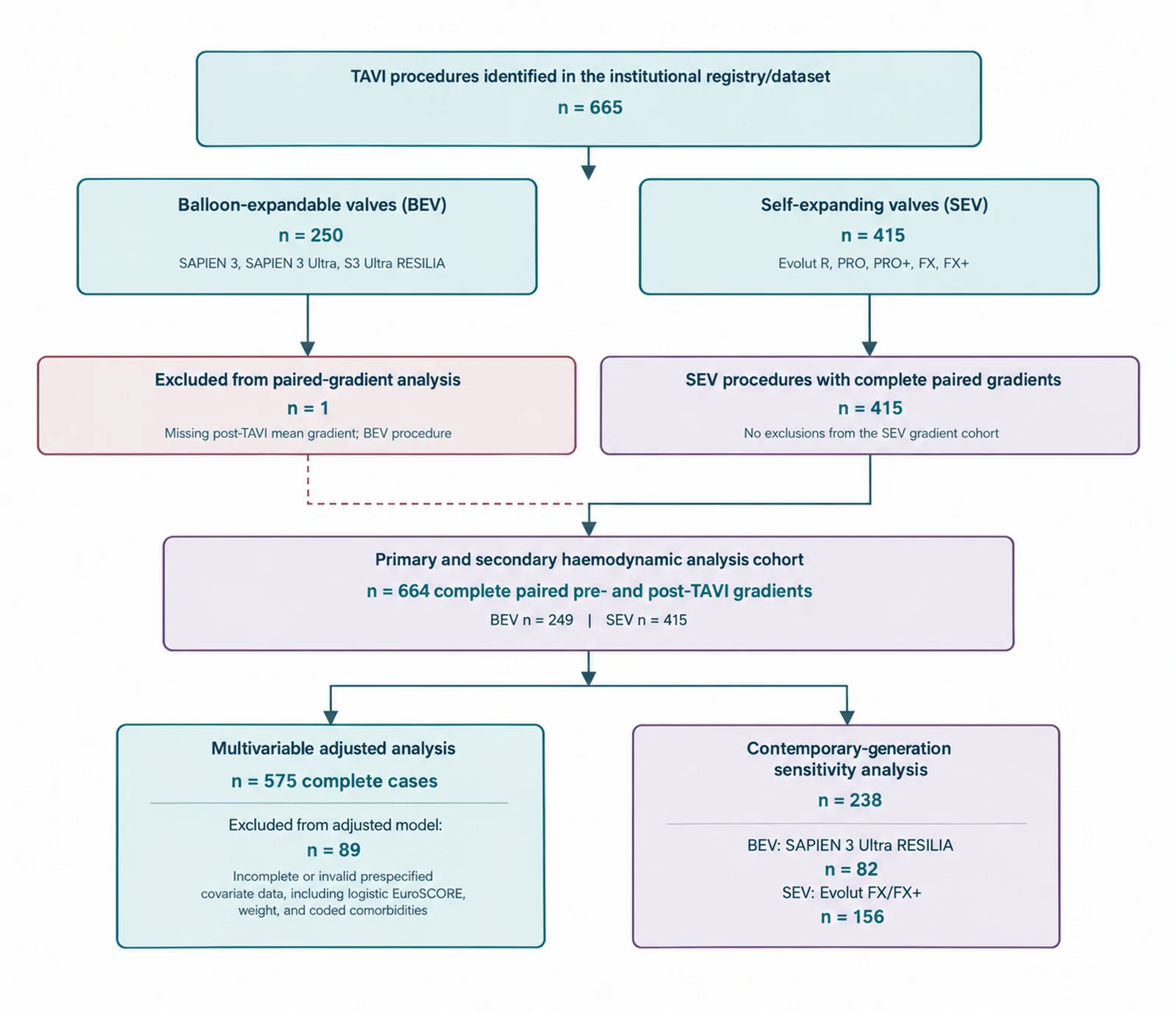
Study population and analysis flow Flow diagram of TAVI procedures included in the retrospective haemodynamic cohort and subsequent analysis populations. Of 665 procedures, 250 used BEVs and 415 used SEVs. Paired pre- and post-TAVI echocardiographic gradients were available for 664 procedures. The adjusted analysis included 575 complete cases. The contemporary-generation sensitivity analysis included 238 procedures. BEV: balloon-expandable valve, SEV: self-expanding valve, TAVI: transcatheter aortic valve implantation

Baseline characteristics

Table 1 summarises baseline characteristics. Weight was similar between the BEV and SEV groups (77.0 (67.0-90.0) vs 76.1 (66.0-89.0) kg; two-sided Mann-Whitney U test: p = 0.381), as was the logistic EuroSCORE (9.5 (7.0-13.9) vs 10.7 (7.4-15.8); p = 0.071).

**Table 1:** Baseline clinical and haemodynamic characteristics Values are n/N (%) or median (IQR); n values below continuous summaries indicate the number of non-missing observations. Denominators vary because of variable-level missingness. BEV: balloon-expandable valve, COPD: chronic obstructive pulmonary disease, IQR: interquartile range, PCI: percutaneous coronary intervention, SEV: self-expanding valve, TIA: transient ischaemic attack

Characteristic	BEV (n = 250)	SEV (n = 415)	p-value
Female sex	106/250 (42.4%)	200/415 (48.2%)	0.147
Weight, kg	77.0 (67.0-90.0) n = 241	76.1 (66.0-89.0) n = 404	0.381
Logistic EuroSCORE	9.5 (7.0-13.9) n = 217	10.7 (7.4-15.8) n = 366	0.071
Diabetes	66/249 (26.5%)	91/414 (22.0%)	0.184
COPD	29/250 (11.6%)	59/414 (14.3%)	0.329
Previous PCI	34/249 (13.7%)	44/413 (10.7%)	0.246
Atrial fibrillation	90/250 (36.0%)	109/414 (26.3%)	0.0084
Previous TIA/stroke	40/250 (16.0%)	53/414 (12.8%)	0.250
Pre-TAVI mean gradient, mmHg	41.0 (36.0-53.0) n = 249	45.0 (38.0-56.0) n = 415	0.0023

There were no significant differences in female sex (42.4% vs 48.2%; Pearson chi-square p = 0.147), diabetes (26.5% vs 22.0%; p = 0.184), chronic obstructive pulmonary disease (11.6% vs 14.3%; p = 0.329), previous percutaneous coronary intervention (13.7% vs 10.7%; p = 0.246), or previous TIA or stroke (16.0% vs 12.8%; p = 0.250). Atrial fibrillation was more frequent among BEV recipients than SEV recipients (36.0% vs 26.3%; Pearson chi-square p = 0.0084). The SEV group had a higher pre-TAVI median gradient than the BEV group (45.0 (38.0-56.0) vs 41.0 (36.0-53.0) mmHg; two-sided Mann-Whitney U test: p = 0.0023).

Primary haemodynamic endpoint

Among the 664 procedures with complete gradient measurements, the median early post-TAVI gradient was modestly lower with SEVs than with BEVs (8.4 (5.85-11.63) vs 10.0 (7.93-13.90) mmHg). The median difference for SEV versus BEV was −1.60 mmHg (bootstrap 95% CI: −2.97 to −1.00 mmHg; Mann-Whitney U test: p < 0.001). Figure 2 shows the distributions.

**Figure 2:**
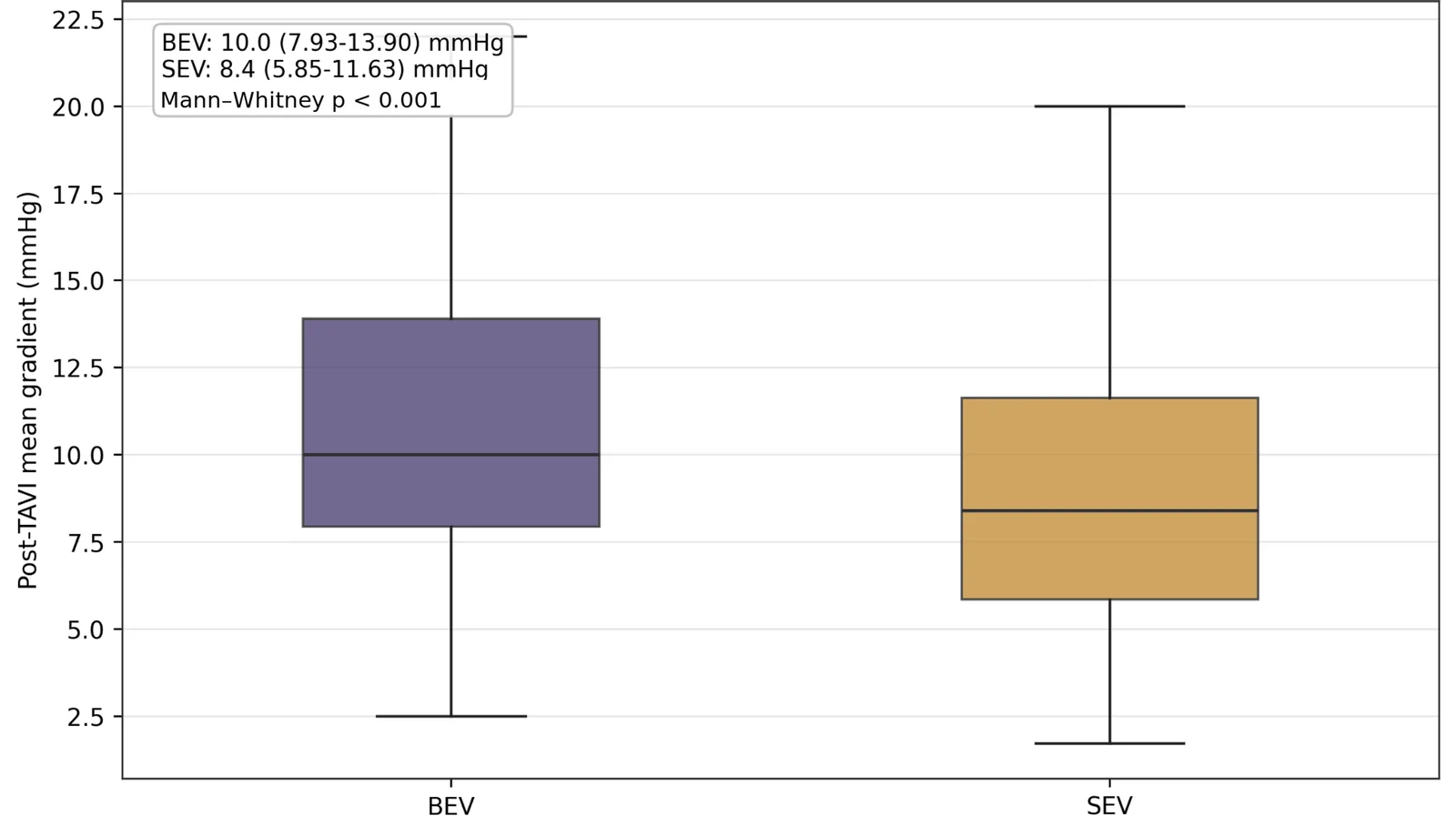
Distribution of post-TAVI mean gradients by valve platforms Box-and-whisker plots show post-TAVI mean gradients for BEVs (n = 249) and SEVs (n = 415). Values are presented as median (IQR). The between-group comparison used the Mann-Whitney U test. BEV: balloon-expandable valve, SEV: self-expanding valve, TAVI: transcatheter aortic valve implantation, IQR: Interquartile range

Secondary haemodynamic outcomes

Paired pre- and post-TAVI analysis showed a significant reduction in mean transvalvular gradient in both groups (BEV: median 41.0 to 10.0 mmHg; SEV: 45.0 to 8.4 mmHg; two-sided Wilcoxon signed-rank test, p < 0.001 for both). The magnitude of gradient reduction was then compared between valve platforms. SEVs were associated with a greater median absolute reduction than BEVs (36.0 (28.4-46.2) vs 32.0 (24.4-41.0) mmHg; median difference, 4.0 mmHg; bootstrap 95% CI: 2.0-7.3; Mann-Whitney U test: p < 0.001). The median relative reduction was likewise greater with SEVs (81.52% (74.42-86.96) vs 76.19% (66.67-81.82); median difference, 5.33 percentage points; bootstrap 95% CI: 3.53-7.05; Mann-Whitney U test: p < 0.001). Because the SEV group had a higher pre-TAVI gradient, these change-from-baseline outcomes were interpreted in the context of this baseline imbalance.

A post-TAVI mean gradient <10 mmHg was recorded in 254 of 415 SEV procedures (61.2%) and 108 of 249 BEV procedures (43.4%). The risk ratio for SEV versus BEV was 1.41 (95% CI: 1.20-1.66; p < 0.001). Gradients ≥20 mmHg were uncommon and did not differ between groups: 25/415 (6.0%) with SEV versus 14/249 (5.6%) with BEV (risk ratio, 1.07; 95% CI: 0.57-2.02; p = 0.831). Table 2, Figure 3, and Figure 4 summarise these results.

**Table 2:** Post-TAVI haemodynamic outcomes Continuous values are median (IQR), and continuous effect estimates are median differences with 95% bootstrap CIs; categorical effect estimates are risk ratios. p-values for independent continuous outcomes are from Mann-Whitney U tests. BEV: balloon-expandable valve, CI: confidence interval, IQR: interquartile range, RR: risk ratio, SEV: self-expanding valve, TAVI: transcatheter aortic valve implantation

Outcome	BEV	SEV	Effect estimate (SEV vs BEV)	p-value
Post-TAVI mean gradient, mmHg	10.0 (7.93-13.90)	8.4 (5.85-11.63)	-1.60 (-2.97 to -1.00)	<0.001
Absolute gradient reduction, mmHg	32.0 (24.4-41.0)	36.0 (28.4-46.2)	4.0 (2.0 to 7.3)	<0.001
Relative gradient reduction, %	76.19 (66.67-81.82)	81.52 (74.42-86.96)	5.33 (3.53 to 7.05)	<0.001
Post-TAVI mean gradient <10 mmHg	108/249 (43.4%)	254/415 (61.2%)	RR 1.41 (1.20-1.66)	<0.001
Post-TAVI mean gradient ≥20 mmHg	14/249 (5.6%)	25/415 (6.0%)	RR 1.07 (0.57-2.02)	0.831

**Figure 3:**
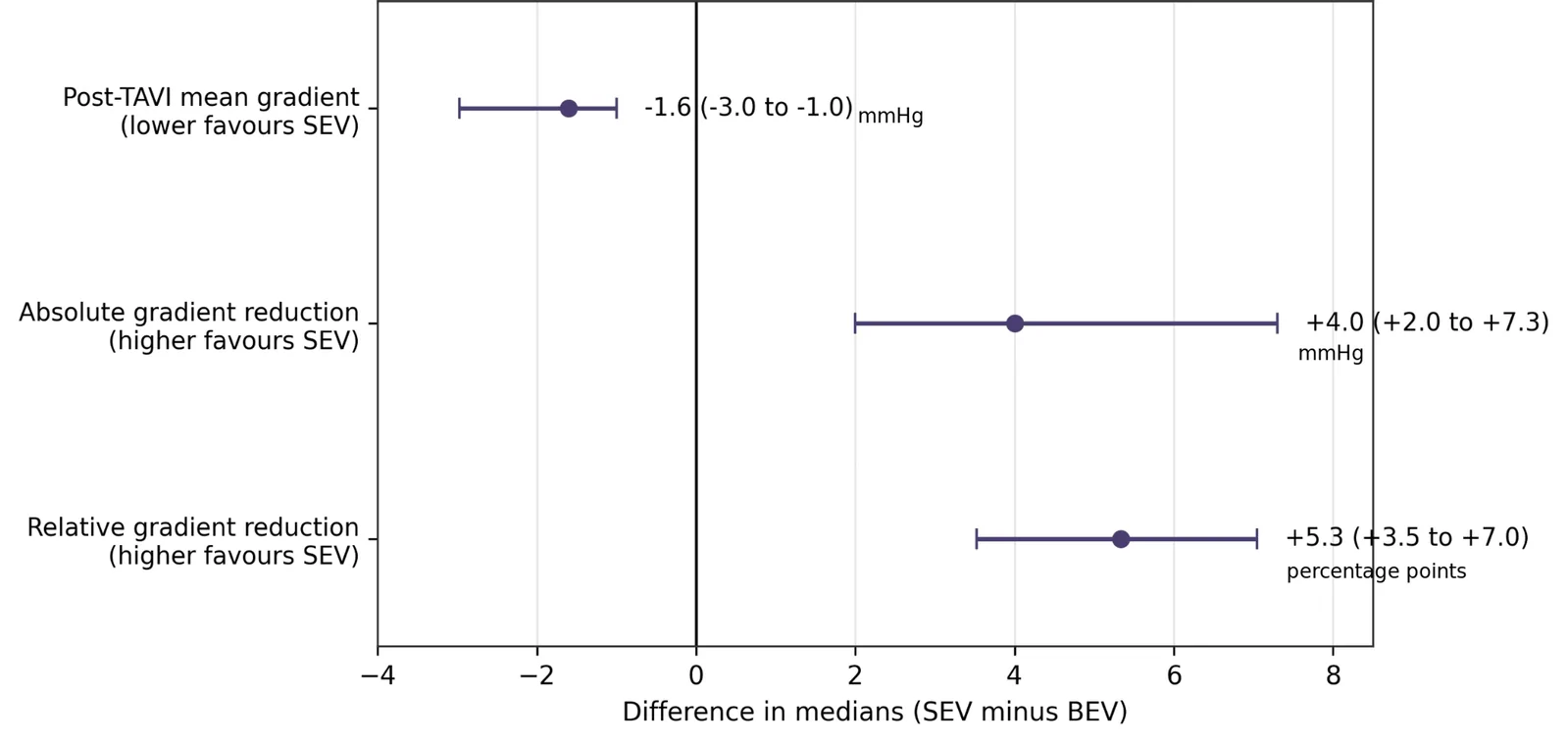
Non-parametric haemodynamic comparisons Differences are reported as SEV minus BEV. The post-TAVI mean gradient and absolute gradient reduction are expressed in mmHg; relative gradient reduction is expressed in percentage points. Points show the median difference and horizontal bars show the bootstrap 95% CI. Negative post-TAVI mean gradient values and positive gradient-reduction values favour SEV. BEV: balloon-expandable valve, SEV: self-expanding valve, TAVI: transcatheter aortic valve implantation, CI: confidence interval

**Figure 4:**
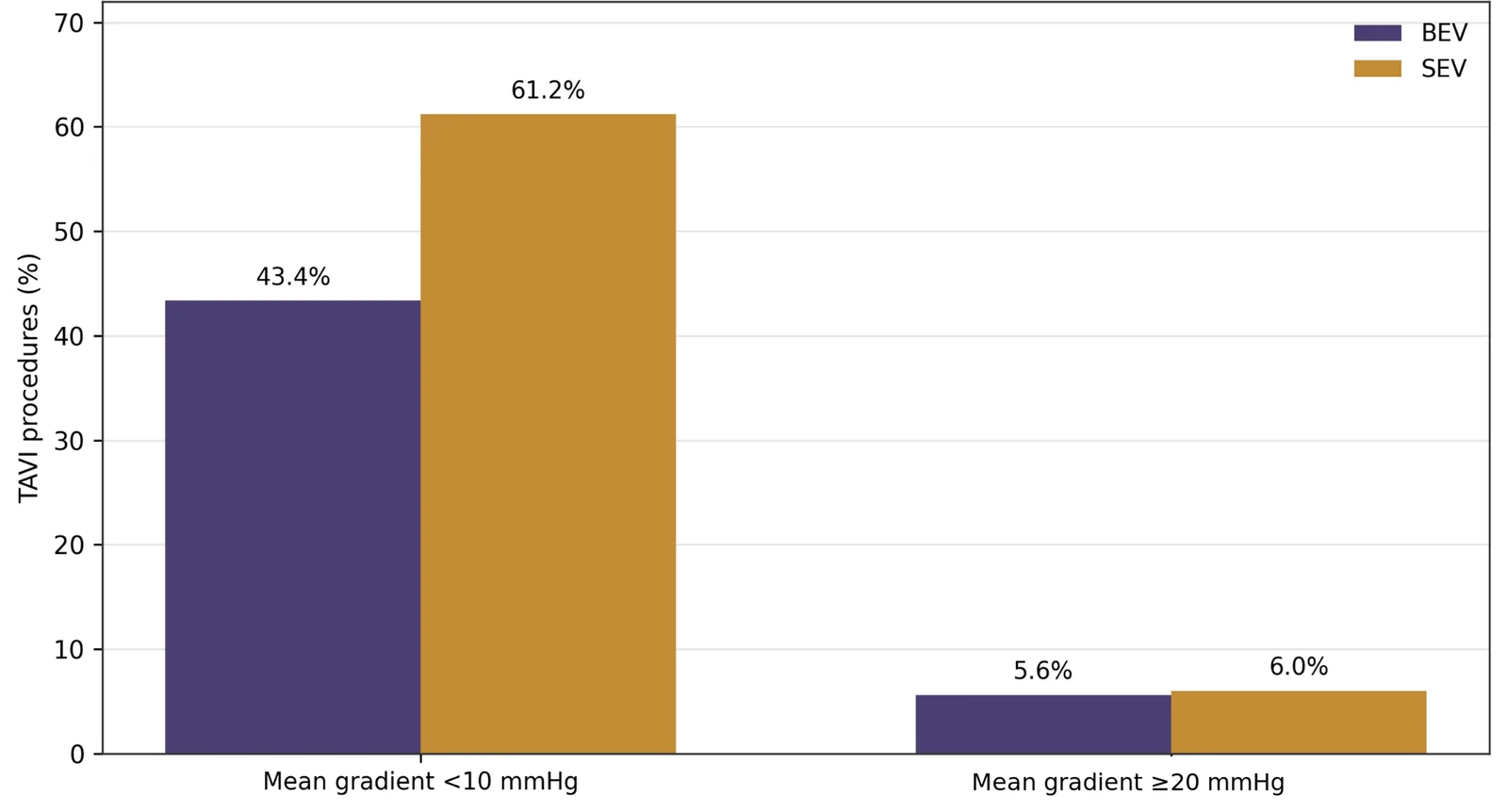
Post-TAVI categorical haemodynamic outcomes by platform Bars show the percentage of procedures with a post-TAVI mean gradient below 10 mmHg or at least 20 mmHg among BEV procedures (n = 249) and SEV procedures (n = 415). SEV procedures more often achieved a mean gradient <10 mmHg, whereas residual gradients ≥20 mmHg were uncommon and similar between groups. BEV: balloon-expandable valve, SEV: self-expanding valve, TAVI: transcatheter aortic valve implantation

Adjustment and sensitivity analyses

After adjustment for pre-TAVI gradient, sex, weight, logistic EuroSCORE, diabetes, chronic obstructive pulmonary disease, previous percutaneous coronary intervention, atrial fibrillation, and previous TIA or stroke, SEV use remained associated with a modestly lower post-TAVI mean gradient. Among 575 complete cases, the adjusted association was −1.48 mmHg (95% CI: −2.42 to −0.55; p = 0.0019). The adjusted association with relative gradient reduction was 3.35 percentage points (95% CI: 0.88-5.82; p = 0.0079). This is demonstrated in Table 3. These estimates represent associations after adjustment for recorded covariates and do not account for anatomical or procedural variables that were unavailable in the dataset.

**Table 3:** Adjusted associations between SEV use and haemodynamic outcomes Models were adjusted for pre-TAVI mean gradient, sex, weight, Logistic EuroSCORE, diabetes, chronic obstructive pulmonary disease, previous PCI, atrial fibrillation, and previous TIA/stroke. Heteroscedasticity-robust HC3 standard errors were used. BEV: balloon-expandable valve, CI: confidence interval, SEV: self-expanding valve, TAVI: transcatheter aortic valve implantation

Outcome	Complete cases	Adjusted effect (SEV vs BEV)	95% CI	p-value
Post-TAVI mean gradient	575	-1.48 mmHg	-2.42 to -0.55	0.0019
Relative gradient reduction	575	+3.35 percentage points	0.88 to 5.82	0.0079

The contemporary-generation sensitivity analysis included 238 procedures: 156 Evolut FX/FX+ SEV procedures and 82 SAPIEN 3 Ultra RESILIA BEV procedures. The median post-TAVI gradient was 7.80 (5.00-11.23) mmHg with Evolut FX/FX+ and 9.05 (6.81-11.27) mmHg with SAPIEN 3 Ultra RESILIA. The direct median difference was −1.26 mmHg (bootstrap 95% CI: −2.38 to 0.51). The two-sided Mann-Whitney U test indicated a nominal rank-based distributional difference (p = 0.020); however, because the bootstrap CI included zero, this analysis was interpreted as exploratory rather than conclusive evidence of a non-zero difference in population medians.

The median absolute gradient reduction was 35.53 mmHg with Evolut FX/FX+ and 31.24 mmHg with SAPIEN 3 Ultra RESILIA, with a median difference of 4.30 mmHg (bootstrap 95% CI: 0.77-8.03; Mann-Whitney U test: p = 0.010). The corresponding median relative reductions were 82.40% and 78.65%, respectively, with a median difference of 3.75 percentage points (bootstrap 95% CI: 1.26-6.25; Mann-Whitney U test: p < 0.001).

A post-TAVI gradient <10 mmHg occurred in 107 of 156 Evolut FX/FX+ procedures (68.6%) and 51 of 82 SAPIEN 3 Ultra RESILIA procedures (62.2%; risk ratio, 1.10; 95% CI: 0.90-1.35; Pearson chi-square p = 0.321). A gradient ≥20 mmHg occurred in 3 of 156 Evolut FX/FX+ procedures (1.9%) and 2 of 82 SAPIEN 3 Ultra RESILIA procedures (2.4%). Because of the small cell counts, we used a two-sided Fisher's exact test (p = 1.000). This contemporary-generation analysis was interpreted as exploratory because of its smaller sample size and limited precision. Figure 5 shows the contemporary comparison.

**Figure 5:**
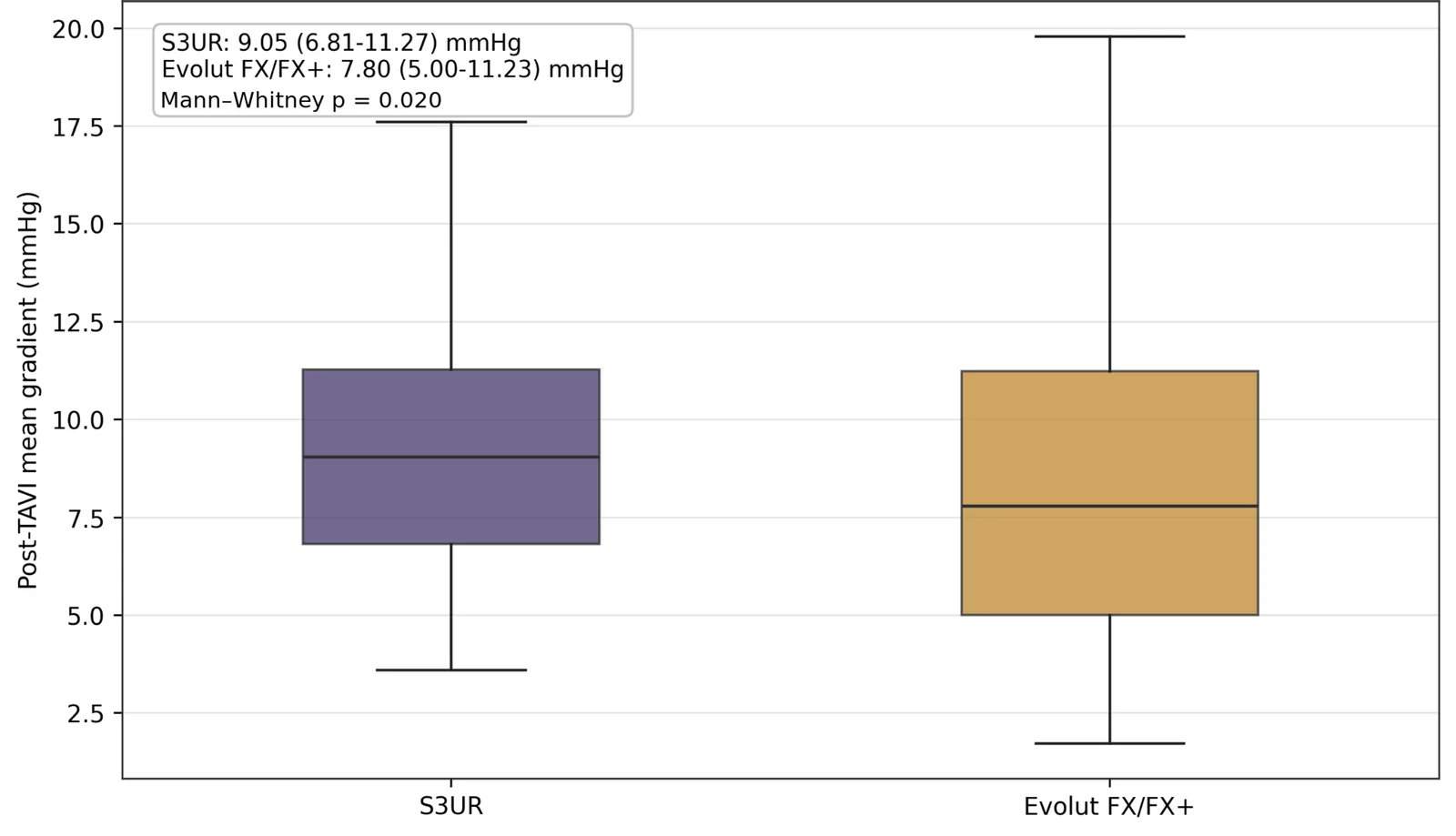
Contemporary-generation sensitivity analysis comparing S3UR with Evolut FX/FX+ Box-and-whisker plots show post-TAVI mean gradients in the contemporary-generation sensitivity cohort: S3UR (n = 82) and Evolut FX/FX+ (n = 156). Values are presented as median (interquartile range). P-values are from the two-sided Mann-Whitney U test. Because the bootstrap CI included zero, interpret the gradient difference as exploratory. S3UR: SAPIEN 3 Ultra RESILIA, TAVI: transcatheter aortic valve implantation, CI: confidence interval

## Discussion

In this retrospective, single-centre observational study, we compared early post-TAVI haemodynamic performance between SAPIEN BEVs and Evolut SEVs in a large, real-world cohort spanning several device generations. Of the 665 individual procedures identified, 664 had complete paired pre- and post-TAVI gradients and were included in the primary haemodynamic analysis. Continuous outcomes were assessed using non-parametric tests and bootstrap CIs, while adjusted associations were examined using ordinary least-squares regression with HC3 robust standard errors in 575 complete cases. SEV procedures had a modestly lower median post-TAVI mean gradient than BEV procedures (8.4 versus 10.0 mmHg; median difference, −1.60 mmHg), and this association persisted after adjustment for measured covariates (−1.48 mmHg; 95% CI: −2.42 to −0.55). Gradients below 10 mmHg were more frequent among SEV procedures, whereas gradients ≥20 mmHg were uncommon and similar between platforms. The contemporary-generation sensitivity analysis was less precise and was interpreted as exploratory because its bootstrap CI included zero.

The observed findings are compatible with known design-related differences between the platforms. Supra-annular leaflet positioning can provide a larger effective flow area for a given annular dimension than an intra-annular prosthesis, offering a plausible haemodynamic explanation for lower gradients with some SEV designs [19]. This mechanism is most relevant when the annulus is small relative to body size, because a smaller functional orifice increases the risk of residual obstruction and prosthesis-patient mismatch [20]. The concept of prosthesis-patient mismatch remains relevant to transcatheter prosthetic performance, although its significance and clinical impact continue to evolve [21,22].

This study's findings are consistent with comparative evidence from earlier and contemporary devices. CHOICE demonstrated platform-level differences in device performance, and a later follow-up showed a larger prosthetic valve area and lower trans-prosthetic gradient with the self-expanding platform [11,12]. Propensity-matched analyses in patients with small annuli and multicentre analyses incorporating annular anatomy have also reported lower gradients with supra-annular SEVs [13-15]. The SMART trial provides recent randomised evidence in a selected small-annulus population, with lower follow-up gradients for Evolut than SAPIEN [17]. The present cohort adds a routine-practice perspective by describing early haemodynamic distributions across several device generations and estimating the magnitude and uncertainty of the platform association in that setting.

The higher baseline gradient in the SEV group is important for interpreting absolute and relative reductions. These change-from-baseline measures combine the pre-TAVI imbalance with the post-TAVI measurement and should not be taken as platform-specific effects in isolation. The SEV group had a 4.0 mmHg higher median baseline gradient, creating greater potential for absolute improvement; however, this baseline difference does not, on its own, explain the lower directly measured post-TAVI gradient, which was the primary endpoint. Adjustment for baseline gradient and other available covariates did not eliminate the observed association. Relative gradient reduction is a useful complementary descriptive measure because it incorporates the baseline gradient. Still, it does not replace the absolute post-TAVI gradient or other validated measures of prosthetic performance.

The threshold analyses provide a clinically interpretable description of the gradient distribution. The greater proportion of SEV procedures with gradients below 10 mmHg suggests a shift toward lower values across the distribution rather than an effect driven only by a small number of extreme observations. Conversely, the low and similar frequency of gradients ≥20 mmHg indicates limited power to detect differences at this uncommon upper-tail threshold. These thresholds describe the distribution of early gradients, but do not replace effective orifice area, indexed measures, Doppler velocity index, flow assessment, longitudinal haemodynamics, or patient-centred outcomes. Their interpretation is therefore best integrated with the broader prosthetic and clinical assessment. The contemporary analysis was smaller and non-randomised, and although it replicated the direction of the primary results, its CI supports a cautious interpretation of its estimate.

The findings of this retrospective, non-randomised, single-centre cohort study should not be interpreted as evidence that one valve platform is universally superior. Post-TAVI gradient, although important, is just one component of valve selection. In practice, clinicians consider post-TAVI haemodynamics alongside annular anatomy, calcium distribution, coronary access, conduction-system risk, vascular access, anticipated future coronary intervention, and operator assessment. The present findings support considering platform-related haemodynamics alongside these factors; they do not justify a platform-specific preference or a clinical decision based on gradient differences alone.

Limitations

The retrospective, non-randomised, single-centre design may be affected by residual confounding, treatment-selection bias, temporal changes, and evolving practice during the 2017-2025 study period. The findings describe associations within this registry and are not causal estimates.

The dataset did not include annular area or perimeter, valve size, body-surface area, effective or indexed effective orifice area, Doppler velocity index, stroke-volume index, implantation depth, left-ventricular function, native calcium distribution, or other detailed anatomical and procedural variables. These factors influence post-TAVI gradients and may have differed between platforms. The adjusted estimates therefore represent associations based on measured covariates rather than causal effects.

Missing observations produced variable denominators and reduced the multivariable analysis to 575 complete cases, excluding 89 procedures. Among these 89 procedures, logistic EuroSCORE was missing in 82, weight in 20, diabetes in 2, previous PCI in 3, chronic obstructive pulmonary disease in 1, atrial fibrillation in 1, and previous TIA/stroke in 1; these categories overlapped. No imputation was performed.

The primary comparison pooled several device generations, introducing hardware heterogeneity. This pooling reflects the prespecified platform-level, routine-practice question but limits generation-specific inference. The contemporary-generation sensitivity analysis included 238 procedures and remained non-randomised; the Mann-Whitney U test and bootstrap interval addressed different quantities, the latter including zero, and the analysis was interpreted as exploratory.

## Conclusions

In this retrospective, single-centre cohort, Evolut SEV procedures were associated with a modestly lower median early post-TAVI mean gradient than SAPIEN BEV procedures (8.4 vs 10.0 mmHg; median difference, -1.60 mmHg). SEV procedures also showed greater absolute and relative reductions from baseline, although the SEV group had higher baseline gradients, making those change measures baseline-dependent. Gradients ≥20 mmHg were uncommon and similar between platforms. The adjusted analysis showed a consistent association after accounting for recorded covariates, while important anatomical, procedural, flow-related, and functional determinants were unavailable. These findings provide a real-world haemodynamic comparison across several device generations; however, their clinical implications require interpretation alongside individual anatomy and other valve-selection considerations. No clinical outcomes or valve-durability measures were assessed; therefore, this study's findings do not establish clinical or device superiority, survival or durability benefit, or a platform-specific valve-selection recommendation. These findings support further prospective evaluation of valve-platform haemodynamics while retaining a patient-specific approach to valve selection based on the full anatomical and clinical context.
